# Animal ethical mourning: types of loss and grief in relation to non-human animals

**DOI:** 10.3389/fvets.2025.1526302

**Published:** 2025-04-28

**Authors:** Panu Pihkala, Elisa Aaltola

**Affiliations:** ^1^Faculty of Theology, Finland & Helsinki Institute of Sustainability Science HELSUS, University of Helsinki, Helsinki, Finland; ^2^Department of Philosophy, University of Turku, Turku, Finland

**Keywords:** grief, animal studies, ecological grief, extinction studies, companion animal, wildlife, animal rights, disenfranchised grief

## Abstract

People can feel various kinds of loss and grief in relation to non-human animals. This has been increasingly studied in relation to pets and companion animals. Recent explorations of ecological grief include wildlife loss, and emerging studies observe grief among veterinarian professionals, zoo personnel, and animal researchers. People can mourn many kinds of animals, including farmed animals, but there is a need for more research on the topic. In this interdisciplinary article, we draw attention to various forms of what we call animal ethical mourning: grief experienced as a consequence of moral commitment to animals. We chart many new aspects by applying Pihkala’s recent framework of Ecological Sorrow (2024) into three case examples: companion animal grief (including pets), wildlife grief, and farmed animal grief. We find many kinds of loss and grief in relation to the case examples, and we propose two new terms for socially contradicted forms of animal ethical mourning: “contested grief” and “contrapuntal grief.” The results are useful for anyone who either experiences animal ethical mourning or wishes to provide more understanding for it in societies. The findings can also inform practices in workplaces which include animals.

## Graphical abstract

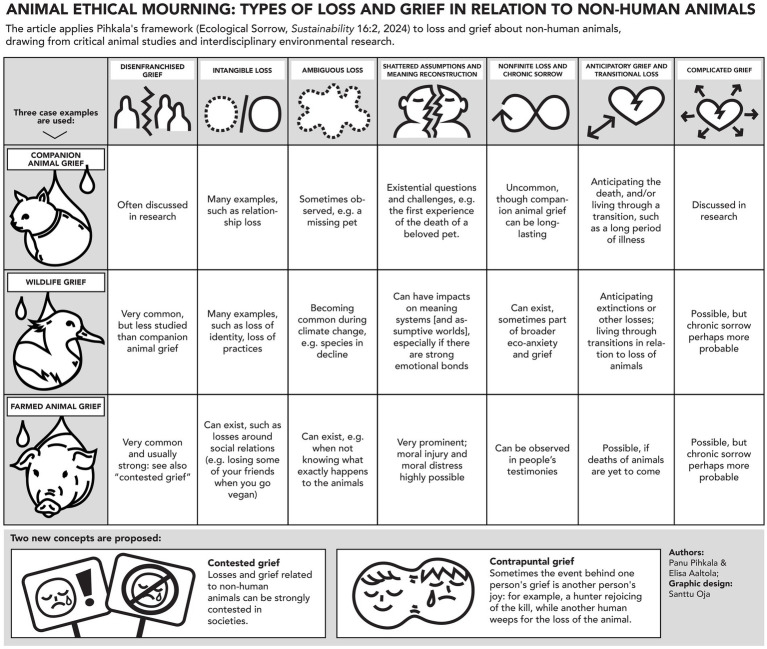

## 1 Introduction

The fates of non-human animals can cause many kinds of feelings of loss and grief (1, 2). Some of these have been recognized in research, most notably grief after the loss of a companion animal or a pet [for reviews, see (3, 4)]. However, as a whole, this grief and mourning has largely remained unnoticed and unrecognized (5, 6), especially in relation to loss of wildlife (7, 8) and the deaths of farmed animals (9, 10). In terminology from grief research, there has been disenfranchised grief (11, 12), but often in very powerful forms.

In this article, we study what we call “animal ethical mourning”: grief experienced as a consequence of moral commitment to animals (13–15). This is closely related to a broader framework of mourning the non-human, which is most often called “ecological grief” (16, 17) and sometimes “environmental grief” (18). While there are writings which study animal ethical mourning as part of ecological grief [e.g., (8, 19–21)], there are many aspects of it which deserve more attention and more careful study. Socially conflicted issues such as meat eating and hunting are difficult topics for ecological grief research, and interdisciplinary, critical animal studies can contribute to further understanding of these phenomena.

Studying animal ethical mourning is important for many reasons. Grief has an ethical aspect: what people grieve reveals their values and emotional attachments, and processes of grief can include moral transformation (22, 23). Several philosophers and animal rights activists have argued that it is moral to grieve the suffering of non-humans (6, 14, 24), and especially if such suffering is caused by humans, there is a need to engage with the issue of responsibility and the dynamics of guilt. Guilt, however, often complicates grief processes (25), and studies on animal ethical mourning need to engage with guilt dynamics (26, 27). Understanding animal ethical mourning is important also for reasons related to well-being and functionality: unrecognized, unprocessed and/or traumatic grief can cause many complications (28, 29), not the least for animal advocates (30) and others seeking to make a change. Other closely related professions and positions include veterinarians, veterinary nurses, those working with animal research, and various animal care workers and volunteers [see, e.g., (31–33)].

In this article, we apply Pihkala’s recent framework (34) of various types of loss and grief in ecological grief into animal ethical mourning. We have chosen three cases as focal points of our analysis: companion animal/pet loss, wildlife loss, and grief over the treatment of farmed animals. These three cases are all important both ethically and in relation to the wellbeing of both humans and other animals. The three cases also reflect different kinds of human-animal relations. We use terms “companion animal grief,” “wildlife grief” and “farmed animal grief” to depict these.

We focus here on the grief human animals feel over nonhuman animals, and leave the topic of grief felt by the latter to further research, as well as the topic of “multispecies mourning” (35, 36), felt and shared across species (13). Furthermore, there are other affective states, such as trauma and shock [9, 37, e.g., (38)], which can entwine with animal ethical mourning, but we leave these mainly outside the scope of this article. Two collections have been especially relevant for us: Margo DeMello’s *Mourning Animals* (24), which explores how one may grieve for different kinds of animals (companion animals, road kill, farmed animals, and so forth), and Johnston’s and Probyn-Rapsey’s *Animal Death* (24), which offers historical and cultural insights into the matter. Closely related fields of study include critical animal studies, extinction studies, grief and bereavement research, and psychological studies of professionals who work with animals such as veterinarians.

Especially in footnotes, we will make observations about animal-related grief felt by various professionals who work with captive animals. These include laboratory workers/researchers (31, 33, 39) and zoo workers (40–42). These kind of professions, whether done for a living or as a volunteer, include complicated ethical relationships to animals. These people have major responsibility for the lives, deaths, and well-being of the animals, and there are various ethical issues and debates related to the use of animals for research (laboratories) or for public display (zoos) (43, 44). Based on earlier research, it is evident that many people working in these facilities develop significant emotional connections to the animals, and that there is grief included when the animals suffer, die, or are transferred. Scholars argue that this grief needs more attention, and we agree, but because of the complexity of these matters, we mostly leave this for future research.

## 2 Applying grief research to the topic

### 2.1 Types of loss and grief

Grief and bereavement research has focused mostly on cases where a close human person has died or is predicted to die (45, 46). When applied to ecological grief and animal ethical mourning, there is a need to move forward from anthropocentrism [Kevorkian (47); as argued already by Windle (48)], and to keep in mind that many aspects of grief research have focused classic bereavement cases. What helps in this is that in recent years, other losses than death have started to gain more attention in grief research, and a framework of “non-death loss” has been generated (49).

Loss and grief are closely related, and in people’s minds, these two are sometimes conflated. However, grief scholars point out that it is important to be able to make distinctions between these two. There can be various types of loss and various types of grief [e.g., (50)]. Not everyone feels the same things to be losses, and a loss may evoke sadness in only some of its witnesses. Scholars have explored “a psychology of loss” in order to pay respect to the complexity of phenomena around loss (51). Distinctions have been made by scholars between primary loss and secondary loss: one loss (primary) can lead to another (secondary) (52).

**Figure 1:**
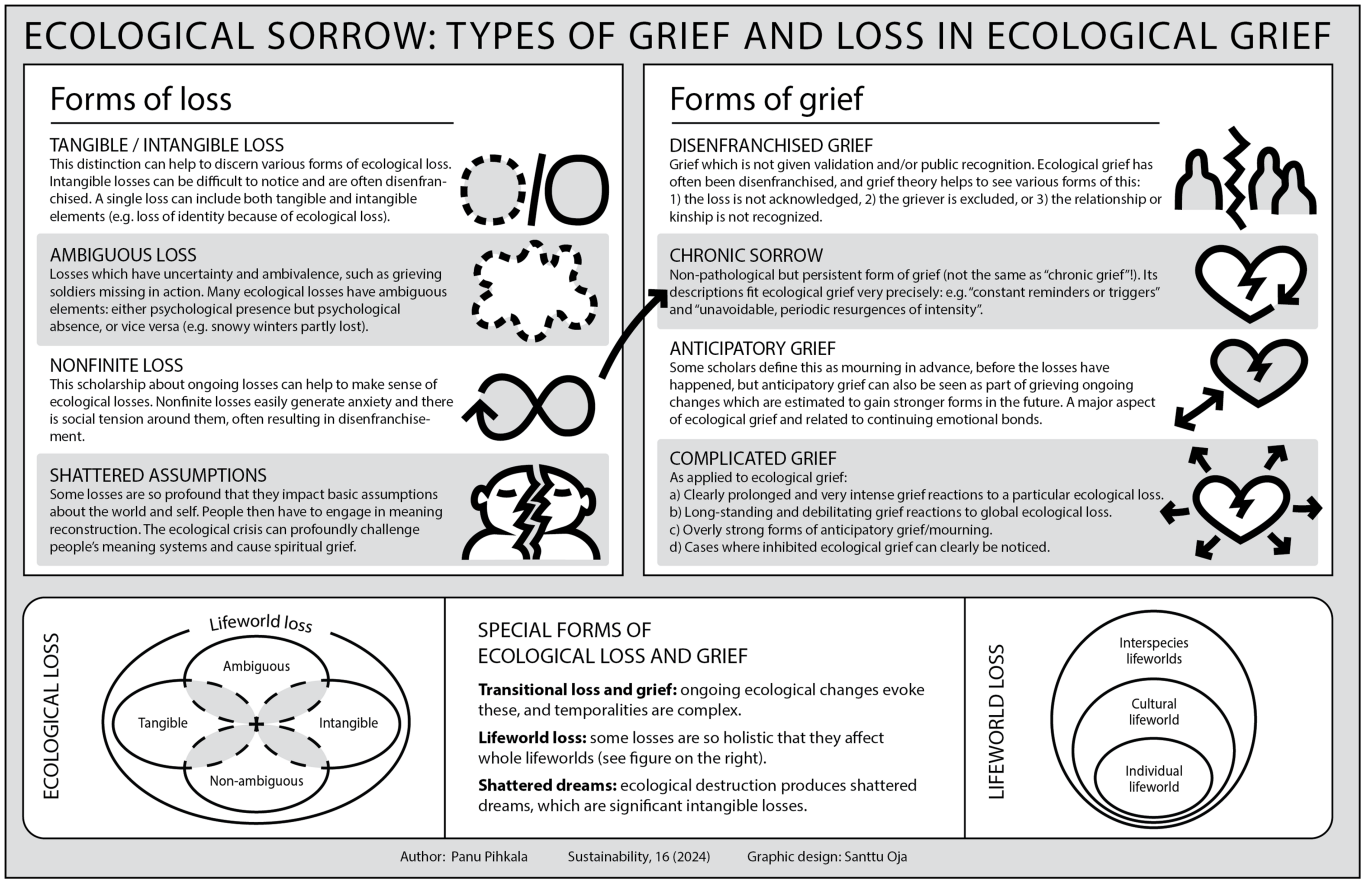
Types of loss and grief as applied to ecological grief (34).

Based on a review of earlier research and integrative work, Pihkala (34) discussed four types of loss frameworks and four types of grief frameworks in relation to ecological loss and grief. In addition, three new formulations about types of loss and grief were proposed to be helpful for understanding the scope and nuances of ecological loss and grief. These are seen in Figure 1.

As seen in the Figure, certain types of loss often lead to certain types of grief: for example, nonfinite loss often leads to chronic sorrow. Many of these types of loss have been found to increase the potential of disenfranchised grief, such as intangible losses and ambiguous loss. Only some of these types of loss and grief have been studied before in relation to animal ethical mourning, and we will review earlier research in the sections which focus on these various types.

There has been a long-standing and often intense discussion in grief research and public policy about what kind of grief counts as somehow problematic (29, 53). Various terms for such grief have been proposed, and sometimes they reflect the proposed defining features, such as “prolonged grief” (28). In this article, the term “complicated grief” is used as a broad name for forms of grief which are somehow extraordinarily difficult.

### 2.2 Factors affecting loss and grief

Grief research shows that many kinds of factors shape people’s responses. Eminent grief scholar Worden (29) offers a framework of seven “mediators of mourning” in order to map these. Some of these factors are related to how losses happen and how people react to the losses. Some factors are related to grieving styles and the person’s other characteristics which shape grieving. In the background, there are numerous kinds of social, cultural, and political factors which shape grieving [for a review, see (54)].

**Figure 2:**
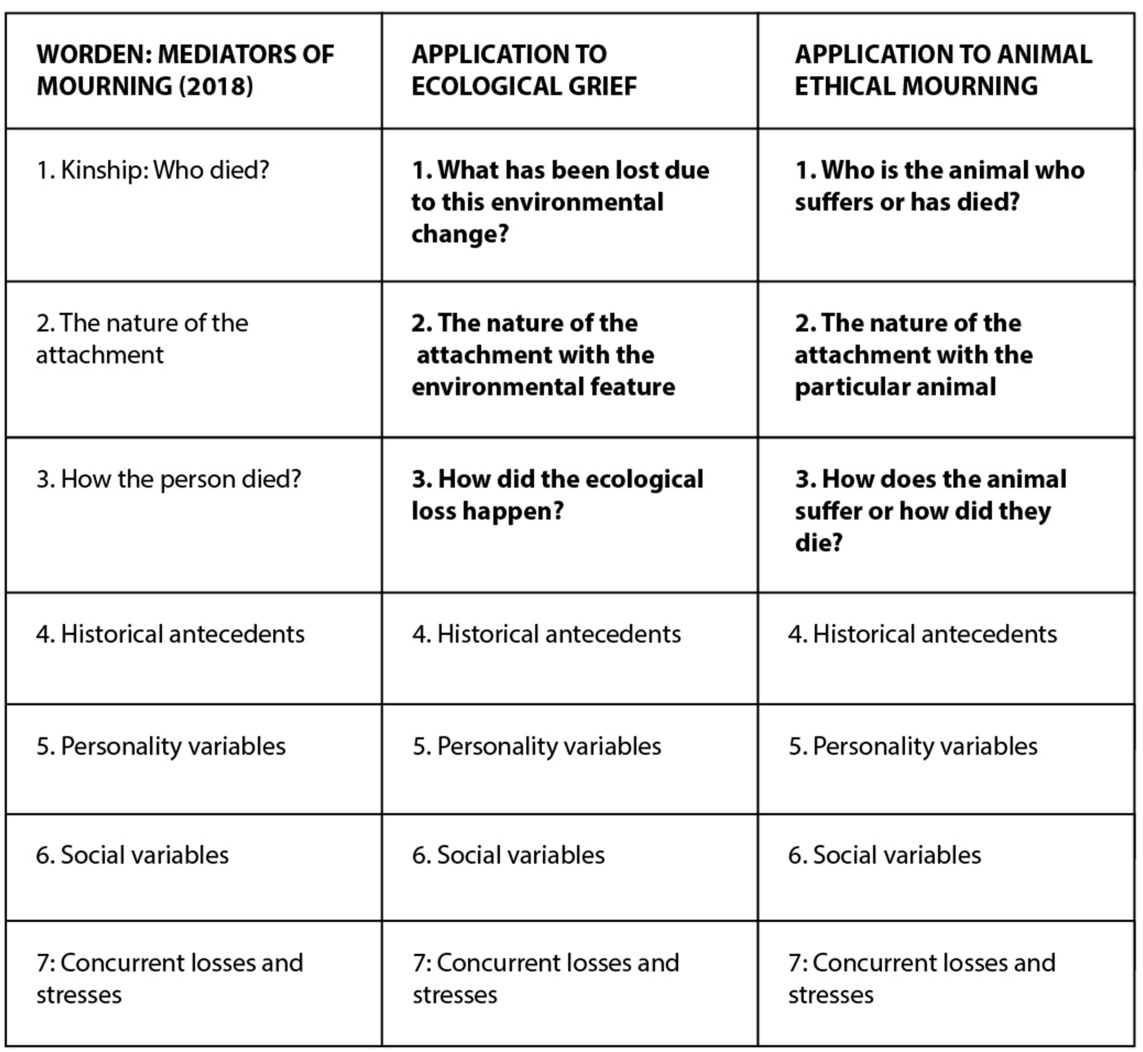
Applying Worden’s mediators of mourning into ecological grief and animal ethical mourning. Major changes marked with emphasis.

The following Figure 2 depicts Pihkala’s (34) application of Worden’s anthropocentric mediators of mourning into ecological grief and our application of the content to animal ethical mourning [see also (55)].

It would be good to add “ecological variables” to Worden’s list, also in cases of human deaths. Worden also discusses various other factors related explicitly to the loss:

- Proximity
- Suddenness or unexpectedness
- Violent/traumatic deaths
- Multiple losses
- Preventable deaths
- Ambiguous deaths
- Stigmatized deaths (29, pp. 61–65)

These factors shape reactions to all three our case examples of animal ethical mourning. Some of them have been observed in research about companion animal/pet loss (4), and it should be noted that they feature heavily in reports by zoo workers about the animal losses which they felt to be the most difficult (42, p. 5). However, these factors are also very relevant for wildlife grief and farmed animal grief: there are a lot of violent/traumatic deaths, multiple losses, and preventable deaths in those spheres [e.g., (9)]. In other words, there are profound grievances, not only grief. We will make further observations about this below.

### 2.3 Dynamics of grief

Grief can also be suppressed or repressed, and it is not always visible or even acknowledged by the griever. People grieve differently and it would be problematic to presuppose similar grief reactions to given types of losses [e.g., (56, 57)]. Therefore, conscious forms of grief (grief that the griever is aware of) can take a multitude of forms. Next to them, also unconscious grief (grief that remains hidden to the griever) deserves attention. Terms such as “inhibited grief” and “delayed grief” have been used to study related phenomena (29, pp. 143–145). Scholars have observed complexities in people’s reactions to ecological grief, pointing out to “inability to mourn” [e.g., (58)]. One possible framework for this is melancholia (59).

These dynamics are important for animal ethical mourning. First, one may manifest such mourning in a multitude of ways, not all of which are apparent to others. Second, animal ethical mourning may remain suppressed, which is arguably common particularly in relation to societally disavowed varieties of animal ethical mourning, such as farmed animal grief (6, 24). There can be silence around wildlife grief [e.g., (60)], and scholars have observed complexities also in relation to speaking about pet/companion animal grief [e.g., (3, 61)].

Grief can have many temporalities, sometimes simultaneously. It can be related to past losses, ongoing losses and/or foreseen future losses. Ecological grief can be related to any and/or all of these temporalities (34, 62). It is important to analyze temporalities in relation to various cases of animal ethical mourning, because temporalities affect mourning: it is significant whether the loss has already happened or is predicted to happen. As will be seen below, some forms of animal ethical mourning combine many temporalities.

**Figure 3:**
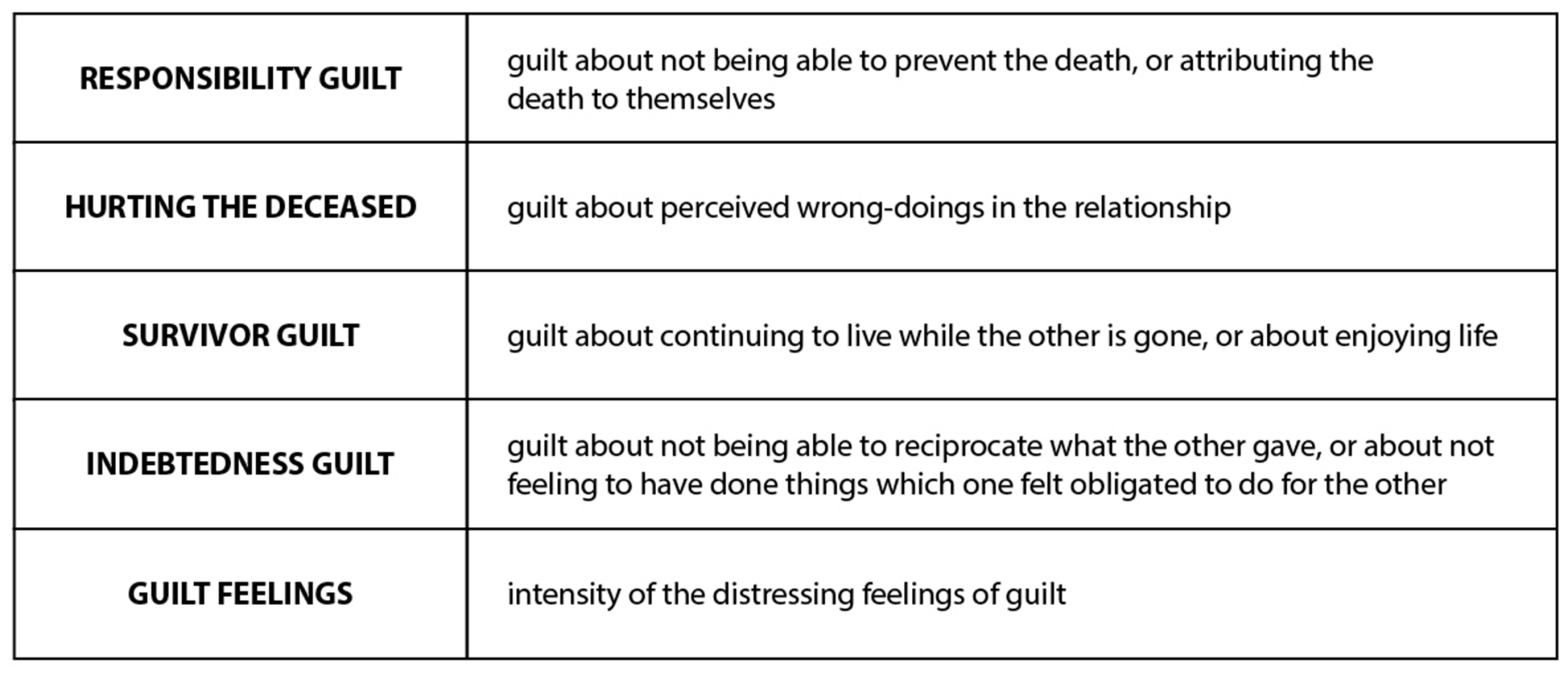
Bereavement guilt scale (63).

In research about grief and bereavement, guilt has been found to be a very common element in people’s processes (25, 57 chapter 6, 29, pp. 21–22). People can feel guilt for the loss itself, or for not having been good enough to the person who is now gone. While some people are more prone than others to feel guilt, it is a standard thing to think about one’s own relation to the loss and to the one who has been lost or is suffering. Various common aspects of grief in bereavement are captured in the Bereavement Guilt Scale (63), which contains five elements as seen in Figure 3.

These kinds of guilt seem to be present in cases of animal ethical mourning, and the commonality of feelings of guilt is noted for example in research on pet/companion animal loss [e.g., (3)]^1^. However, up to our knowledge, the Bereavement Guilt Scale and categorization has not previously been applied to the topic, and we will make observations of it in our analysis.

### 2.4 Outcomes of grief

In 20th Century grief research, an influential connotation was the idea of decathexis: that mourners would need to cut emotional attachments to lost ones, so that they could move on in life. However, especially since the 1990s, there has been a major movement in grief theory towards what is commonly called continuing bonds. Mourners very often wish to continue emotional attachments with the lost ones, in a new form, for example via memorialization (64, 65). This has been observed also in studies about animal ethical mourning, especially in relation to companion animals (4, 66), but also for example in relation to extinctions (67).^2^

As mentioned, grief often feels difficult and it can lead to depression or other forms of complicated grief. However, it can also be a process of profound growth. Various frameworks and terms have been used to study this in grief research. Post-traumatic growth (PTG) is a well-known framework (68), but also, e.g., adversarial growth has been suggested to apply to cases without trauma (69). Growth via grief can include many factors, such as enhanced appreciation of life, better treatment of others, and greater determination to take care of personal limits and well-being. These dynamics are important also in relation to animal ethical mourning, especially since such mourning can foster growth in our attitudes concerning and treatment of nonhuman animals [e.g., (8)]. Scholars have started to study PTG in relation to companion animal grief [e.g., (70)] and among veterinarians (71), but much remains unstudied.

## 3 The three case examples

The chosen three cases – companion animal grief, wildlife grief and farmed animal grief – reflect various types of relations between humans and other animals. In companion animal grief, there are strong emotional bonds and often also personal attachment at play. In a given culture, certain kinds of animals are more common as companions, such as dogs and cats in Western cultures. For instance, dogs are able to express affection toward their companions. Moreover, studies show that human beings can form deep attachment to companion animals – at times deeper than toward their conspecifics (72–74). In other words, we can feel love toward also nonhuman creatures, and feel that some of those creatures reciprocate our feelings (26, 75). This love renders grief felt when losing our animal companions more pronounced [e.g., (4)].

There can be various kinds of wildlife loss, and sometimes also combinations of wildlife loss and human losses (76, 77). The strength of emotional bonds differs greatly between various contexts. For example, for indigenous peoples a certain wildlife species can be immeasurably important in a cultural, relational, and also psychological sense, such as in the case of rendeer for Sámi people (78). For urban dwellers, the decline of many wildlife species can happen almost unnoticed, but there are also people who are sensitive to it (79), as manifested already by the seminal book “Silent Spring” by Rachel Carson (80). For people who have special connections with a type of an animal, such as an ardent birder (or ornithologist) with birds, the loss of that species may be devastating [e.g., (81)]. Visible wildlife loss, such as roadkill, evoke feelings of sadness in some, while others drive by (or over) the dead carcasses; again, there may be people who are specially affected by the loss, such as animal care workers whose duty it is to pick up wounded animals and try to care for them (82). Some people dare to witness via arts (83) or spirituality: the indigenous people studied by Chao (35) sing songs of mourning when they encounter roadkill animals, testifying to kinship, and Veldkamp (84, p. 60) tells of an annual Buddhist mourning rituals for roadkill in Korea.

On average, however, wildlife is not as close to contemporary people in industrialized societies as companion animals are to those who live with them, but wildlife grief has still been a prominent part of for example grief about wildfires intensified by climate change (85, 86), and it can be a cause for developing an environmentalist/animal rights activist identity (15). Moreover, many professions can make one vulnerable to wildlife grief, including being a wildlife researcher (87) or an animal care worker (32).^3^

The concept of vicarious grief is helpful here. It refers to grief experienced because of someone else’s loss or a faraway loss (88). There can be vicarious grief in relation to wildlife loss (and in some cases in relation to deaths of companion animals of other people): for example, many people have grieved when they see images of animals burned in wildfires for example in the Amazon or in Australia, even while they are far away. And in farmed animal grief (9), vicarious grief is very prominent.^4^ Although there are some activists who see and feel the suffering of mistreated farmed animals firsthand,^5^ others grieve for them from a distance – if at all (6). Indeed, most people are not allowed to enter agricultural sites, even if they wanted to, and also recording evidence from such sites, intended for public use, is often forbidden.^6^ As a result, farmed animal grief remains arguably rare.

Farmed animal grief concerns a vast and ongoing phenomenon, and thus it has certain similarities with wildlife grief: both happen on a global scale and are caused by human activity. However, farmed animal grief has some distinct elements, unusual in other contexts. Most importantly, it can concern substantial yet systemic cruelty toward animals, including castration without sedation and forced separation of young from their mothers. We suggest that these moral violations make farmed animal grief especially difficult: it is hard to witness the extreme moral wrongs committed by human beings to other animals. By consequence, one may end up feeling what we have elsewhere called “misanthropic melancholia,” a prolonged process of grief combined with misanthropic sentiments (34) (there are many kinds of factors at play here: for example, when a person has special affinities toward a certain species, observing the suffering caused to members of that species can feel especially grueling).

It is to be noted that we focus on grief caused by industrial animal farming and on various ethical violations. When animals are raised on a much smaller scale, for example by agrarian families, there can be feelings of grief felt by at least some members of the family when the animal suffers, goes missing, or dies [e.g., (89)]. There is a long history of human experiences here in agrarian societies, but we will not venture deeper into this topic.

We now move on to discussing many types of loss and grief categorized by Pihkala (34) in relation to animal ethical mourning.

## 4 Disenfranchised grief

In grief research, it has been noted that certain forms of grief can be difficult for communities and societies. One such form is disenfranchised grief (DG). It refers to cases where grief is not “openly acknowledged,” “socially validated,” and/or “publicly mourned” (11, 12). Grief philosopher Ratcliffe summarizes the core of DG: “Others fail to acknowledge or legitimate one’s grief, in ways that affect one’s access to processes that shape grief’s trajectory” (90, p. 211).

The reasons for DG can be manifold, but often there is both ethical complexity and lack of empathy behind these instances (91, 92). The particular loss at hand may be felt to be too difficult to handle psychologically, or there may be for example economic conflicts at play: if recognition of the loss would mean that a person or a group would have to give up some kind of hedonic pleasure or economic advantage, there is often resistance. Because social support is elementary in relation to constructive engagement with grief, lack of it is problematic and sometimes devastating. It is little wonder, then, that DG has been connected with complicated or prolonged grief (see section 2 above).

Numerous scholars have noted that disenfranchised grief in relation to companion animal grief is frequent (93, 94). For example, one is usually expected to return to work and function as normal, even if one’s long-term nonhuman companion is severely ill or has died – the society does not legitimize the severity of grief felt in such instances [for case examples, see (95)]^7^. There is less scholarship about DG and the other two case examples. However, e.g., James Stanescu and Kathryn Gillespie have emphasized that farmed animal grief tends to remain wholly socially disavowed (6, 24), which also renders it regrettably rare; and scholars such as Kevorkian (18) have observed DG in relation to wildlife. Researchers have observed the prevalence of DG also in zoos [e.g., (42)]^8^ and laboratories [e.g., (96)], and inside a given organization, there may be varying opinions about the importance of grieving in public [in general, see (97)].

**Figure 4:**
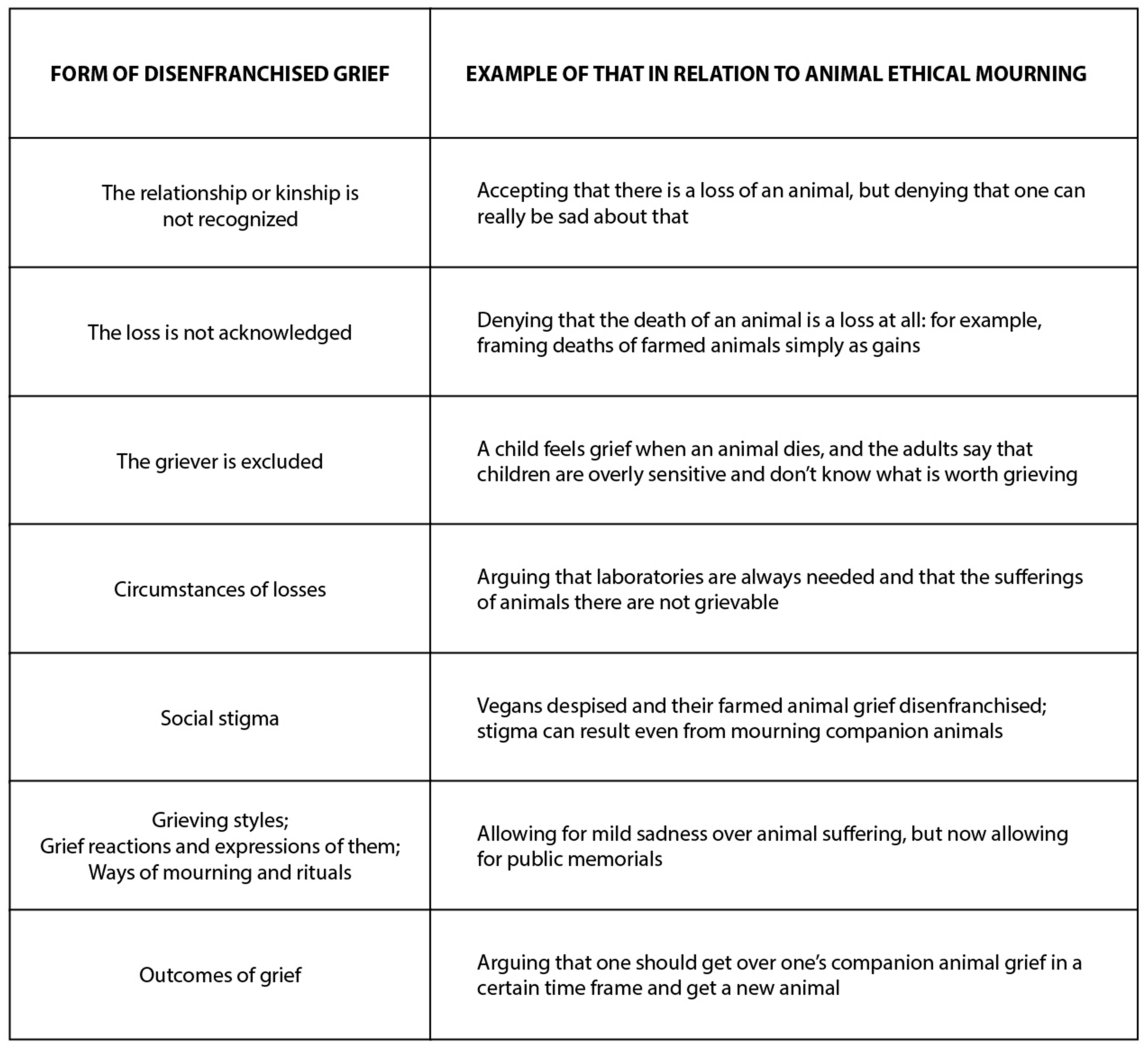
Applying forms of disenfranchised grief into animal ethical mourning.

In the following, we apply the categorizations of various kinds of DG dynamics (11, 98) into the case examples, pointing out that there can be quite different degrees of DG in relation to them. We are especially interested about the latter two case examples, since there has been less research about DG and them, and because there seems to exist powerful forms of DG, or even worse, in relation to them. Before more nuanced analysis, Figure 4 gives an overview.

Some of these distinctions have been discussed in relation to companion animal grief (3, 4, 61, 94, 99), but in general, they would deserve more nuanced attention in the context of animal ethical mourning and its different sub-varieties. In relation to companion animals, the most common forms of DG seem to be Doka’s two first categories: a lack of recognition of the depth of attachments and relationships, combined with not acknowledging the severity of the loss. These can range from total disregard to various forms of derogation or belittling: a typical form would be commenting that “it’s only an animal, you can always get a new one” [e.g., (100), p. 179]. These kind of comments manifest a strongly anthropocentric worldview and an empathic failure. Redmalm (61) points out that pet/companion animal grief challenges human exceptionalism and social disapproval of such grief can be an effort to maintain that exceptionalism. He notes that those who grieve their animal companions can themselves partly belittle their grief in social situations, which would account for self-disenfranchised grief [for the concept, see (11)].

In relation to wildlife and farmed animal grief, even deeper problems emerge, amounting to what grief scholars Corr and Attig have called ethical and political failures in DG (91, 98). Whilst companion animal grief is at least partly familiar to many, these other two varieties concern stronger deviance from the type of anthropocentric norms customary in most contemporary cultures. As Cohen and Clark observe: “depending on the animal’s species, those who are grieving may experience a response that reflects a further hierarchy of loss, such as, ‘I could understand being so upset if it were a dog, but a bird?’” (99, p. 405).^9^

Therefore, wildlife and farmed animal grief are often culturally contested. We argue that this is motivated by the desire to avoid uncomfortable moral issues, such as one’s own culpability in the death and suffering of nonhuman animals. Indeed, as a method of resisting environmental and animal ethical concerns, others may question the validity of the grievers and question their relationships and kinship with non-human animals [e.g., (6)]. For example, those concerned with nature loss may be called “tree-huggers,” and those concerned with animal rights issues may be called “grass munchers.” DG can be combined with these kind of uses of power (9). Pike (15) observes how animal rights activists may testify to kinship between species and how grief can be a means to express and maintain such bonds (p. 420), thus resisting DG at least in their own spheres.

“Circumstances of the loss” is one category named in DG scholarship, and this seems highly relevant for wildlife and farmed animal grief. Much wildlife loss and suffering of farmed animals is caused by industries deemed socially acceptable by the current majority of people in industrialized societies. The locations and circumstances of losses are thus bound with contradictions and DG, which may partly explain why wildlife and farmed animal grief are often contested.

Several research articles have studied the dynamics of DG among people who work with animals. For example, Marton et al. (101) observed DG dynamics that animal care workers have experienced [see also (102)], and Englefield et al. (82) pointed out that Australian wildlife carers would need more social support for their DG in relation to witnessing deaths of roadkill animals. Bexell et al. (87) discuss DG amidst conservation professionals, also pointing out that there may be structural failures in environmental research and activism organizations to counteract DG and support professionals, echoing the observations by researchers of animal-related grief in zoos [e.g., (40, 42)].^10^

Pallotta (100) observes that mourning farmed animals can be the target of profound DG, and as a result, the mourners may experience “profound alienation from the mainstream culture and dominant social norms,” and suffer from the “lack of a socially sanctioned outlet for mourning the billions of animals who remain hidden in the machinery of society” (p. 181). Brooks Pribac (9) notes the various degrees which may happen in DG: “human mourners of nonhuman animals, whose grief is usually not recognized, not admitted, and oftentimes even derided, hence falling under the category of disenfranchised grief” (p. 193). DG scholars mention stigma as a related factor, and this appears pertinent also in farmed animal grief [similarly Cohen and Clark (99)]. Grieving for animals can produce stigma, and sometimes this can be motivate individuals to disenfranchise their own grief (4). Stigmatization’s effect can be intense: Stanescu (6) has pointed out that even ardent animal rights advocates can end up, due to social pressures, hiding their own grief.

This leads to an important topic: some dynamics around animal ethical mourning are so full of contradictions and they include such problematic uses of power, sometimes including ostracizing and bullying, that it may be asked whether the literal concept of DG does justice to them. We will return to this in the last section of the paper, where we explore concepts such as “contested” and/or “contrapuntal” grief.

## 5 Intangible loss

The distinction in grief research between tangible and intangible loss is relatively recent, but its substance has been studied for a long time [e.g., (103)]. Tangible losses are more easily noticeable, if people only pay attention. Intangible losses, on the other hand, are characterized by “lack of physical signs or something obvious to the casual observer” (104, p. 239). Examples include the following: loss or change in identity or sense of self; loss of connection to others; loss of social status, and loss of meaning, faith or hope. Intangible losses are often disenfranchised because of the lack of physical objects (104). Pastoral psychologists Mitchell and Anderson created terms for various kinds of intangible losses in the 1980s, without using the concept itself. They mention for example “relationship loss” and “role loss” (105), and these can clearly happen in animal ethical mourning.

### 5.1 Companion animal grief and intangible loss

*“I felt a part of me gone”* (a person after the death of their companion animal (106, p. 132).

The concept of intangible loss itself has not been used in research about companion animal grief, up to our knowledge, but there are many implicit observations of intangible losses in relevant literature. For example, Pallotta (100) found that those who lose a companion animal “typically experience the loss of a dependable source of unconditional emotional support and more quotidian, yet still disruptive, changes, such as the loss of daily routine involved in caring for the animal” (p. 180). She uses the concept of secondary loss, but the concept of intangible loss helps to see further nuances in losses.

Losing a pet or companion animal can cause many kinds of intangible losses. An example is loss or turmoil of identity [(107), pp. 4–5; (61); “The Death of A Dog,” in Rhees (108)]. People can lose connections to others, for example when a rider loses a horse and is not anymore part of the riding community; this can also result in losses related to social status [see also Schroeder (109)]. Caring relations can be lost in many ways: the care and comfort that the animal companion has provided is now lost, and on the other hand, loss of the possibility for the human to be a caregiver of the animal can be a difficult “role loss” (110). Overall, losing a pet or companion animal can result in tangible and intangible losses so deep that they near shattered assumptions and/or changed meaning systems (99, p. 401, 111).

### 5.2 Wildlife grief and intangible loss

…What should we be without

the dolphin’s arc, the dove’s return,

These things in which we have seen ourselves and spoken? (112).

Wildlife grief can also include various intangible losses. Such losses can cause turmoil of identity, both individually and collectively, and the amount of this is connected to the felt significance of the wildlife. For example, for Inuits who have an intricate relationship with caribou, the losses caused by diminishing caribou and from not being able to hunt them cause many kinds of intangible losses, including losses related to identity as not only hunters but as people, and status-related losses to hunters (113). On the other end of the spectrum, a person opposed to hunting can suffer grave intangible losses due to witnessing wildlife being killed, including losing their belief in the human ability for animal ethical regard, and the potential exclusion from their community (if they live in a rural setting). An environmentalist can feel intangible losses for a similar reason. A whole category used in ecological grief research, “grief associated with disruptions to environmental knowledge systems and resulting feelings of loss of identity” (16), is bound with intangible losses. This can apply to a number of groups, ranging from indigenous hunters to environmentalists and animal rights advocates.

To put it simply, tangible losses of wild animals cause intangible losses to people who need them for sustenance or have environmental and/or animal ethical concerns. Krause (20) writes about the sadness caused by vanishing or diminishing sounds of animals; for him, that tangible loss causes intangible losses about relationality and aesthetics. Sometimes the resulting losses are so strong that they result in loss of meaning [e.g., (113)]. Another aspect is related to wildlife which is taken care of in shelters or ends up in zoos; people living and working with these animals can suffer various kinds of intangible losses when something negative happens to the animals [e.g., (40)]^11^.

### 5.3 Farmed animal grief and intangible loss

Loss and grief are intertwined in complex ways in this case. There can be profound intangible losses concerning beliefs and assumptions, e.g., about human morality. A person may have believed their whole lives that societies ensure high welfare to farmed animals, and after realizing this to be untrue, their trust in such societies and human morality can suffer a severe blow. Grieving for farmed animals can spark changes in identity and habits, and these can create or intensify intangible losses related to social relations. For example, becoming vegan can cause disputes in one’s social setting, and lead to feelings of being an outsider, as vegans are often socially stigmatized (114); these are severe intangible losses connected to identity, self, status, and social relations.

As mentioned earlier, we focus in this article on farmed animal grief by others than farmers, but it should be mentioned that losing animals may engender various kinds of intangible losses to farmers, as discussed by Chur-Hansen (89, p. 19). These include, among others, possible role loss, loss of family traditions, and loss of a sense of being a successful farmer. Chur-Hansen warns of PTSD symptoms and profound feelings of guilt and/or shame in these situations.

## 6 Ambiguous loss

Ambiguous loss as a concept focuses on losses that include major unclarity and lack closure. The concept has been developed especially by the grief scholar Boss, who makes a distinction into two possible types of ambiguous loss: the cause of the loss may be “physically absent but psychologically present,” as with soldiers missing in action, or “physically present but psychologically absent,” as with people who have severe dementia (50, 115).

Ambiguous loss is difficult to engage with because of the unclarity, and the situation is often made worse by the common tendency to disenfranchise grief in relation to these kind of losses (the loss may be socially categorized as mild due to its unclarity, which entwines with lack of social recognition). Ambiguous loss also commonly lacks rituals and other community practices, which makes it more challenging to deal with Boss et al. (116). Intangible losses may be ambiguous, and non-death-losses are often prone to include ambiguity (117).

The type of ambiguous loss where a related object is physically present but psychologically absent might be extended to ecological grief by reframing it as physical presence while something essential about the object of loss is gone; an example would be the mourning of a forest which has lost biodiversity and thus something of its very essence (34). This reframing allows one to perceive also various types of animal-related losses as ambiguous.

Boss and other grief scholars have formulated recommendations for coping with ambiguous loss. A list often used by Boss is: “Finding meaning, Adjusting mastery, Reconstructing identity, Normalizing ambivalence, Revising attachment, and Discovering new hope and purpose for life” (50). It follows that these kinds of practices should be studied in relation to animal-related ambiguous loss.

### 6.1 Companion animal grief and ambiguous loss

A clear example of ambiguous loss is a companion animal gone missing (89). This type of loss and the resulting grief is recognized in several materials about animal-related grief [e.g., (118)] and discussed briefly in a recent review (4), where only three studies were found which deal with the topic. Those studies focus primarily on impacts of natural disasters, where pets have gone missing and people experience ambiguous loss. In these studies, people reported higher psychological distress because of ambiguous losses. Walsh (189, p. 488) notes that ambiguous pet loss can cause conflict among family members, because some of them may still wish to hope, while others wish to grieve the loss as final.

An example of physical presence but psychological absence would be a pet or companion animal who starts to suffer from the diminishment of their mental abilities and thus is not the same individual as they used to be – compare dementia or Alzheimer’s disease in humans with Canine Cognitive Dysfunction in dogs [e.g., (119)].

### 6.2 Wildlife loss and ambiguous loss

The framework of ambiguous loss seems to be very important in relation to wildlife loss, because of the difficulty of knowing whether a declining species will be completely lost, either from the local environment or via extinction from the face of Earth. It may be nearly impossible to know whether a total loss has already happened or not, or whether some members of the species still survive somewhere (79, 120).

The framework of ambiguous loss has been mentioned in pioneering studies of ecological grief [e.g., (16)], but it would seem beneficial to apply it more specifically to wildlife loss. For example, a recent study has observed:

*Conservation professionals were profoundly affected by grief from the losses of individual animals, as well as by the extinction of species, which were caused by anthropogenic harms. Findings also showed that conservation professionals were ill prepared to cope with the emotional toll of their work and were forced to build resilience in the face of the unprecedented extinction crisis.* (87)

Whilst the researchers do not mention ambiguous loss, it appears relevant to the case. In another study, Wrigley (120) explicitly engages with the ambiguity related to an emblematic species facing extinction, namely the Scottish wildcat, and points out that “The death of a species creates a much less ambiguous response than its potential loss.”^12^ It seems to us that ambiguous loss in relation to species decline is intimately connected with anticipatory grief/mourning, which is discussed more below.

### 6.3 Farmed animal grief and ambiguous loss

The dynamics of ambiguous loss in relation to farmed animals is in need of further research. For example, there may be ambiguity about the fates of certain animals, if those animals are taken into production units where outsiders cannot go. This would amount to physical absence with psychological presence. On the other hand, the category of physical presence but psychological absence might be applied to cases where an animal, which one has known from when she was young, is transferred to a production unit and eventually loses much of her earlier vitality. Physically, the animal is there, but her essence is gone (cf. a person with brain damage).^13^ On a more general level, one can grieve for pigs and cows despite the fact that they exist in vast numbers: here, one grieves for the way they are treated, and how this treatment prevents them from flourishing as creatures.

## 7 Meaning reconstruction and shattered assumptions

Losses can affect people’s emotional bonds with both the lost “object” (a person, a non-human animal) and with those still present [e.g., (121, 122)]. Moreover, losses can affect people’s meaning systems: schemata and beliefs which guide people’s behavior and tell of their values and meanings in life [see, e.g., (123)]. Sometimes this can have a powerful, even shattering impact [e.g., (124)].

Several terms and frameworks have been used in grief research about these kind of dynamics. Trauma researcher Janoff-Bulman has produced an influential framework of “shattered assumptions.” She points out that traumas are generated by their impact on fundamental beliefs and assumptions, which challenges a person’s feeling of safety (125). Much earlier, influential grief researcher Parkes wrote about the role of “assumptive worlds” in grief: mourners often have at least some of their assumptions about the world questioned, and need to engage in a “psycho-social transition” (57, 126, pp. 188–209). Using related but distinct terms, grief philosopher Attig writes about the need to “re-learn the world” in grief processes [e.g., (124)].

Some scholars prefer to use terminology of “existential crisis” in relation to these kind of issues, while others specify meaning-related crises [e.g., (127, 128)]. Eminent grief scholar Neimeyer and his colleagues have developed a framework of “meaning reconstruction,” which emphasizes the need of mourners to engage with changes in their life meanings. Neimeyer and colleagues have written a lot about this practice, and they often use narrative methods to enable people to process changes in meanings and life narratives [e.g., (46, 129, 130)]. Deep growth may be inherent in these kind of processes, in addition to pain [e.g., (131)].

These kind of deeper dynamics of grief have been studied significantly more in relation to companion animal grief than in relation to the two other case examples of this article, and thus we will focus on the latter two. In many texts about companion animal grief, emotional attachment and continuing bonds are often emphasized (132). Some scholars mention meaning-related dynamics and Neimeyer’s work (133). For example, in a recent review of pet bereavement and coping mechanisms, the possibility of a “crisis of meaning” is mentioned in the context of religious coping; however, the wider dynamics concerning, e.g., shattered assumptions are not discussed (4).

### 7.1 Wildlife grief

For indigenous peoples who have strong involvement with wildlife species, the loss of these species and the related interactions can be shattering (134). Such losses strongly affect their meaning systems and count as “lifeworld loss,” a comprehensive loss of a way of living (34). There may also be shattered dreams, which is a major intangible loss and connected with meaning systems [in general, Bowman (135); in ecological grief, Pihkala (34)].

Also among non-indigenous peoples, loss of wildlife can have vast impacts on meaning systems and assumptive worlds, if the wildlife in question holds significant meaning in the first place (8, 136). An example is the impacts of loss of birds for people who care about them, as Mark Cocker testifies based on his experiences in Britain:

*For statistics and columns of figures do not begin to express the effects of the changes at a personal and interior level. For some people, agricultural intensification has triggered an emotionally charged, even visceral response, at the root of which is a baffling confrontation with local extinction and loss of meaning. The effect is powerful enough to alter an individual’s personality and their entire view of life.* [81; see also (137), pp. 105–107]

### 7.2 Farmed animal grief

The dynamics of shattered assumptions are especially significant in the case of farmed animal grief. As a few scholars have observed (9, 138, 139), the scale of the related animal suffering is so enormous, and the character of the atrocities can be so cruel, that if one faces this reality empathically, it will have an inevitable impact on one’s assumptive world [for case examples, see (30, 140)]. Worden’s (29) distinctions of types of deaths (see section 2 above) are useful here: there are numerous violent/traumatic deaths, multiple losses, and preventable deaths. As we discuss elsewhere, when faced with the scale of suffering and death of farmed animals, one can undergo a loss of belief in our very humanity, and mourn how the meanings central to many, including moral ideals, have been failed by our current, industrial farming and the significant harm it causes to other species (13). Many scholars have observed that in addition to vicarious grief (see section 2 above), vicarious traumas can be generated (9, 37), linked to traumatic grief [for that concept in general, see (141)].

In a recent text, Pribac mentions Janoff-Bulman’ framework of shattered assumptions as relevant to farmed animal grief. The three fundamental assumptions – which are closely related to basic psychological needs – which Janoff-Bulman puts forward are:

- The world is benevolent – generally speaking it is a good place and most people are kind;
- That the world is meaningful, things make sense and happen for a reason; and
- That the self is worthy – by and large individuals perceive themselves as good, capable and moral” [description by Brooks Pribac (9); see also Janoff-Bulman (125)].

As Pribac observes, all these three may be strongly affected by realizing the fates of farmed animals. How to believe that the world is benevolent, that human beings are kind and good, and that things happen for a sound reason, when witnessing the large-scale suffering human beings inflict on farmed animals? For some people, the amount of animal testing adds to this anguish. The assumptive world (57) is profoundly disturbed: one may suddenly question one’s previous beliefs, and even undergo a fundamental sense of meaninglessness. Moreover, emphatic and / or ethically aware people can wrestle with difficult issues related to responsibility and potential guilt. Hansson and Jacobsson (38) discuss this process under the rubric of “re-engineering of affective cognitive repertoires”.

Additional potential impacts are moral distress and moral injury. The concepts refers to psychological distress after having done or having witnessed actions that violate core moral beliefs and norms (this may include distress about a perceived failure to prevent harmful unethical behavior). Moral distress can be seen as the first reaction, which can lead over time to moral injury (71). While these have been discussed the most in relation to soldiers and war veterans, they has also been studied for example in relation to healthcare workers and social workers (142), and veterinarians [e.g., (143–145)]. There are also studies of moral injury in research which includes harming animals (146). It seems evident that coming to know the suffering of farmed animals can produce great moral distress and moral injury, especially if one is personally part of the practices, but there is a lack of research about this in relation to other people than veterinarian professionals.^14^ Some prominent animal rights advocates have observed moral injury and written about the importance to engage with it,^15^ but we could not find studies of moral injury among other people who grieve farmed animals. Yet, it seems evident that the shattered assumptions, mentioned above, can spark a potentially severe sense of moral injury, whereby one begins to feel responsible for not preventing the immense suffering caused to nonhuman animals. Among activists, this can lead to burnout and compassion fatigue (30).

Fundamentally, the resulting meaning reconstruction is an ethical process, as Oliver (139) has argued: “anger imbues multispecies spaces as a destructive and constructive project of renegotiating the world after violent truths of animal abuse are revealed” (Chapter 2).^16^ This could be explored in future studies in relation to post-traumatic growth and adversarial growth: many aspects of it can be easily identified in this context, such as increased compassion and changes in ethical priorities (24, p. 116, 37, pp. 43–45). Post-traumatic growth has actually been studied in relation to moral distress among veterinary professionals in animal care (71).

## 8 Nonfinite loss and chronic sorrow

Because nonfinite loss and chronic sorrow are so intimately related, they are discussed in this same section in this article. Nonfinite loss is a concept that has been developed and used by grief researchers especially since the beginning of the 2000s. It refers to “enduring losses that are typically precipitated by a negative life event or episode where the loss itself retains a physical and/or psychological presence with an individual in an ongoing manner” (147, p. 140, 148). A closely related counterpart among types of grief is chronic sorrow, a framework used and developed since the 1960s.^17^ A major developer of this framework, grief scholar Roos defines chronic sorrow as: “a normal yet profound, pervasive, continuing, and recurring set of grief responses resulting from a loss or absence of crucial aspects of oneself (self-loss) or another living person (other-loss) to whom there is a deep attachment” (149, p. 194).

While there is overlap in the use of these two concepts in grief research, they can be differentiated by observing nonfinite losses as a type of loss and chronic sorrow as a type of grief (117). Nevertheless, scholars of nonfinite loss name many attributes that are actually related to grief processes and not just to loss.

Grief scholars emphasize that there is often disenfranchised grief in relation to nonfinite loss and chronic sorrow (116, 150). Nonfinite losses typically include intangible aspects, such as losses related to identity, which are themselves prone to disenfranchised grief. Some of the intangible losses in nonfinite loss are related to meaning systems and shattered assumptions: the experience of life is now different than what it was before the loss. Roos points out that at the core of chronic sorrow is “a painful discrepancy” between what was supposed to be and what has then become the state of affairs (149, 150). A nonfinite loss continues to manifest itself in one way or another, and chronic sorrow scholars have observed that there can be “unavoidable, periodic resurgences of intensity” and “predictable and unpredictable stress points” (149). Predictable stress points include annual reminders of earlier important engagements with the object of the loss before something happened (150).

**Figure 5:**
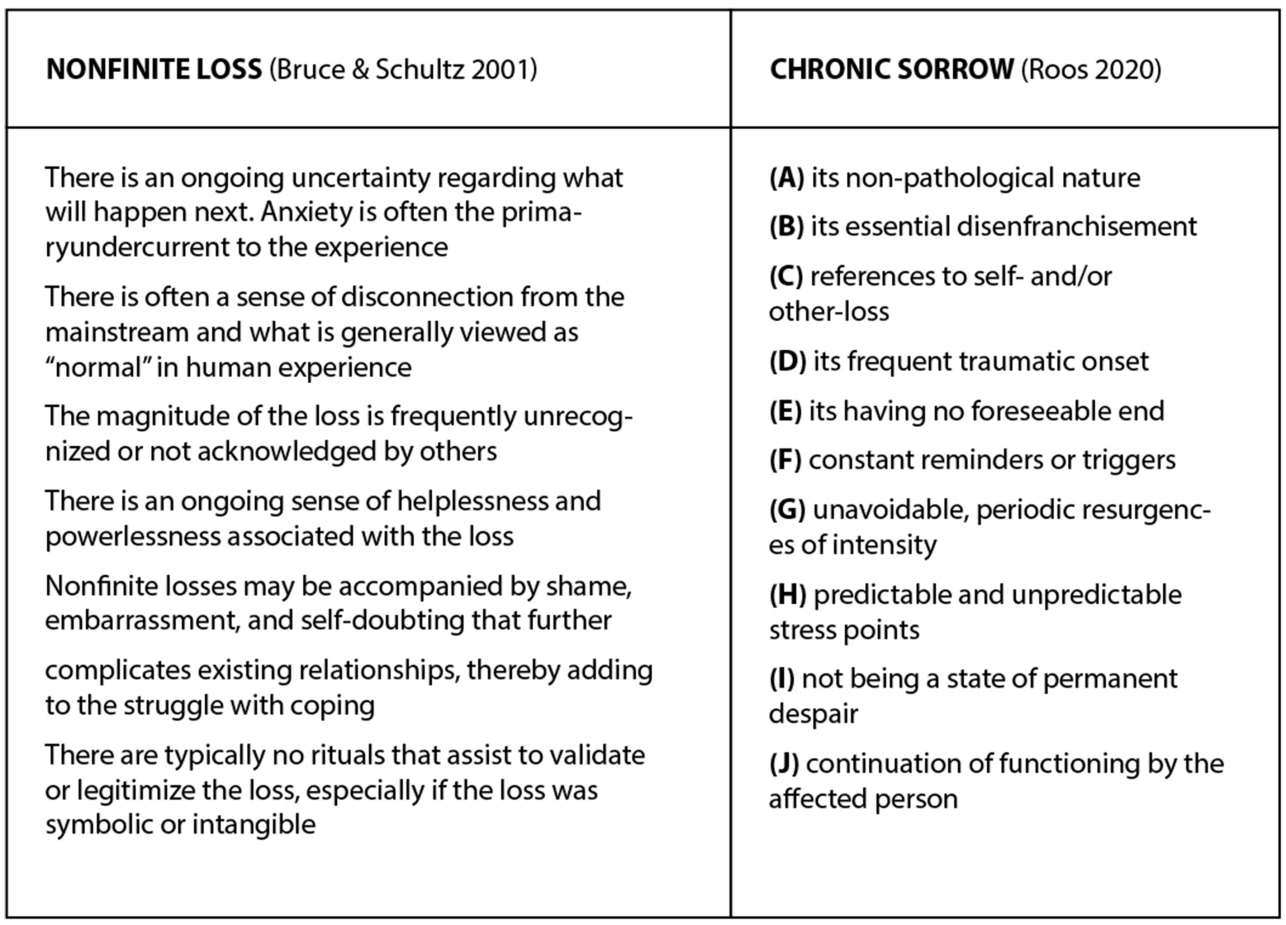
Characteristics of nonfinite loss and chronic sorrow.

These distinctions are useful also in relation to animal ethical mourning. By definition, nonfinite losses are separated from death-related loss (147), and these categories are thus not particularly relevant in relation to companion animal grief.^18^ However, they are highly relevant for wildlife and farmed animal grief. In particular, the common characteristics of nonfinite loss and chronic sorrow, as observed by grief scholars, include much to think about in this context, as manifest in Figure 5.

In written testimonies about wildlife grief, many aspects of nonfinite loss and chronic sorrow are evident. In industrialized societies, only some people have intricate knowledge about wildlife loss, and their feelings may not be recognized or supported by others, leading to disenfranchised grief in response to this nonfinite loss and possible chronic sorrow. For example, many natural scientists and bird-lovers implicitly manifest elements of these phenomena in their writings (81, 87, 58), and knowledge concerning these concepts can help to make sense of their experiences. If extinctions become evident, nonfinite loss and anticipatory mourning turn into tangible loss and possible acute grief, but arguably people who deeply care about wildlife, can experience chronic sorrow even after this process [for examples, see (67)].

In relation to farmed animal grief, the concepts of nonfinite loss and chronic sorrow are pertinent, because the onslaught of farmed animals continues heavily. There is “no foreseeable end” and instead often one is accompanied by “an ongoing sense of helplessness and powerlessness associated with the loss.” For some people, this includes sorrow about animals used in laboratories [for discussion of related phenomena, see (9)]. There are “constant reminders or triggers” and “predictable and unpredictable stress points”: think of a vegan visiting a grocery store and seeing all the meat products. Stanescu has illustrated this poignantly in his depiction of the psychological struggle one may undergo when witnessing signs of animal suffering and death littered all around most cultures (6). The phrase “There is often a sense of disconnection from the mainstream and what is generally viewed as ‘normal’ in human experience” seems to describe common social dynamics in relation to vegans (151), as also illustrated by Stanescu.

There is a need for “repeated adjustment or accommodation” (147). Yet, from a moral viewpoint, such adjustment would be dangerous, as it would help us to bypass the suffering of farmed animals. Thereby, we suggest that nonfinite loss and chronic sorrow can be moral psychological dimensions of animal ethical awareness – manifestations of our care and concern for other species.

## 9 Anticipatory grief and transitional loss

Anticipatory grief is an old topic in grief research (152), but people have differing intuitions about what this phenomenon exactly means. Some think of it as grieving in advance: the old Freudian notion of decathexis means the effort to cut out emotional bonds so that there is no more grief due to loss. Ecological grief scholars have argued that decathexis is an unconstructive solution to environmental loss, because people need to maintain emotional connections with the more-than-human world both for ethical and prudential reasons (62, 153). In grief research, the concept of continuing bonds (see section 2 above) is closely relevant to this.

When analyzed more carefully, three forms of anticipating loss can be discerned:

1. Grieving really in advance, before the loss has happened at all, while it is unclear whether the loss will actually happen.
2. Engaging with a transitional loss where the final outcome is at least partly knowable; for example, dealing with feelings of loss as one becomes older. Death cannot be avoided, but no-one know exactly how one’s old age will go.
3. Engaging with an ongoing loss which has strong anticipatory elements, while the final outcome includes at least some uncertainty. This is the case with the global ecological crisis: the damage is ongoing, more is predicted, but there is also uncertainty.

The first option can be a form of catastrophizing, since it is not known yet whether the loss will happen. The second and third options are combinations of transitional loss and anticipatory loss (34, 154). Grief scholar Rando has constructed a framework of “anticipatory mourning,” thus using a slightly different term, and depicts a complex process “of mourning, coping, interaction, planning, and psychosocial reorganization that are stimulated and begun in part in response to the awareness of the impending loss of a loved one and the recognition of associated losses in the past, present, and future” (52, p. 24).

Anticipatory grief and mourning seem to be common possibilities in all three of our case examples. With companion animals, the situation is as with human companions: one may start to grieve in advance the loss of another, whether for a good reason or not (155, p. 171). Classic causes of anticipatory grief and mourning are new information about an illness or reaching a certain age (4, p. 294). What ensues is a combination of transitional loss and possible anticipatory grief and mourning. Many companion animals have shorter life spans than humans, which affects dynamics of anticipatory grief and transitional loss. If a person starts to feel anticipatory grief at a very early point of the companion animal’s life, this can be argued to bring unnecessary difficulties, but at some point the engagement with transitional loss and anticipatory grief will be an important way to prepare for what is to come [in general, see (29), pp. 204–208]. The latter kind of constructive engagement aligns with Rando’s definition of anticipatory mourning. A special situation for anticipatory grief is a process where euthanasia is planned for the companion animal (4) (see their bibliography for more sources). People in various professions, such as veterinarians and animal care workers, may also have feelings of anticipatory grief.^19^

In relation to wildlife grief, the temporalities of ongoing losses and anticipated losses are very crucial. People mourn losses and extinctions that have already happened [e.g., (156, 157)], but in these times of rapid biodiversity decline, there are constantly ongoing losses and lots of anticipated losses [for discussion, see (79)]. As observed above, these may include ambiguous loss, and grieving numerous and powerful losses can be difficult [e.g., (158, 159)]. Furthermore, the probable amount of extinctions and species decline is partly dependent on various scientific estimations, and mourners may be confused between them, causing social disputes.

Also farmed animal grief can be anticipatory. Here, one focuses on what will eventually happen to the animals alive today (mourning, e.g., for the fate of newborn piglets and lambs, or mourning for the eventual slaughter of a cow). Arguably, such anticipatory grief could occur also among farmers in the era of small-scale farming, whereby an owner may have mourned in advance for the day when the slaughter truck arrives [see also (160)]. Further, anticipatory farmed animal grief can concern future generations of animals. It is common in animal advocacy materials to discuss the amounts of animals used in agriculture in the years to come, and such discussion can entwine with anticipatory grief over the suffering and death of future generations of animals.

## 10 Complicated grief

Frameworks about complicated grief, such as prolonged grief, seem much more relevant in relation to companion animal grief than in relation to the two other case examples of this article (4). Grief resulting from wildlife loss and suffering of farmed animals are more akin to chronic sorrow arising out of nonfinite loss: what would count as “prolonged” amidst a growing number of extinctions? There are cases where the manifestations of those griefs are strong, and social support, sometimes even medical support, would be useful. Vicarious trauma and post-traumatic stress can occur [e.g., (37), pp. 161–163; more widely, see (161)]. But there should be caution not to pathologize this type of grief and instead to understand its complex character.

Pihkala (34) proposed four theoretical categories of complicated ecological grief:

- Clearly prolonged and very intense grief reactions to a particular ecological loss.
- Long-standing, strong and debilitating grief reactions to global ecological loss.
- Overly strong forms of anticipatory grief/mourning.
- Cases where inhibited ecological grief can clearly be noticed.

These can be applied to grief about non-human animals in the following way:

- Clearly prolonged and very intense grief reactions to a particular loss related to non-human animals^20^.
- Long-standing, strong and debilitating grief reactions to the global suffering of non-human animals^21^.
- Overly strong forms of anticipatory grief in relation to non-human animal(s).
- Cases where inhibited grief can clearly be noticed after the loss of non-human animal(s).

Much depends on the connotations in which terminology about complicated grief is used. If it is used to depict something gone wrong in a “normal” process of grief, then its usage in grief about wildlife loss and farmed animals is partly challenging. However, if the terms are simply used to describe complex and difficult forms of grief, then they are more readily applicable.

Sometimes animal-related grief is complicated because of interhuman dynamics and problematic human decisions. For example, veterinarian nurses have been observed to feel complications in grief if they have to witness and even participate to questionable euthanasia (162). And disenfranchised grief commonly complicates grieving (3, 89).

Another option is that there is simply too much death and suffering of animals in relation to coping resources and possibilities of mourning. This kind of overburdening has been observed in many studies [e.g., (89)], including ones about animal care professionals [e.g., (32, 102)].

**Figure 6:**
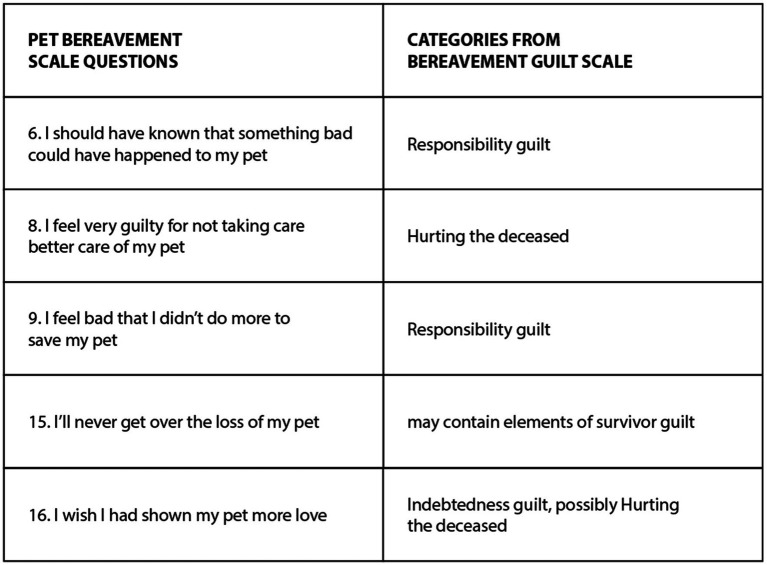
Application of categories from bereavement guilt scale to pet bereavement questionnaire.

Guilt is a very potential complicating factor, and has been studied for example in relation to grief after euthanasia decisions of companion animals [for a review, see (4), pp. 290–291] and other animals (155, 163). General grief research offers possibilities for future work on the nuances of this. For example, it would be good to integrate elements from the Bereavement Guilt Scale (63) to usage of Pet Bereavement Questionnaire (164), and Figure 6 makes a proposal for connections between these two.

Several scholars have discussed the various potentials of guilt and shame in relation to the more-than-human world (27, 165, 166), and overly strong forms of these emotions can complicate grief processes around wildlife grief and farmed animal grief. Guilt can lead to depressive moods and even a desire to punish and damage oneself [e.g., (24), p. 55]. Scholars have noted the complex feelings of guilt or inadequacy which can arise because people are connected in profound ways to social structures and practices which produce animal suffering [24, 167, p. 191; e.g., (158)]. Furthermore, because of anthropocentric norms of grieving, those who mourn animals may feel guilt and/or shame simply because of their grief (99, p. 405), which shows the interconnections of disenfranchised grief with these issues.

Engaging with various dimensions of guilt, for example via the themes in Bereavement Guilt Scale, could help in moving towards the constructive potential in guilt and shame [for that potential, see (165)]. Worden (29) recommends “reality testing” guilt in bereavement, and this seems very important in relation to animal ethical mourning. A trusted person, or a group, can help individuals to think about how justified their feelings of guilt actually are. For example, a child who has internalized culpability for the death of a pet (110), can be supported in realization that such guilt is a grief reaction and not actually a fair assessment of responsibility. In relation to larger ethical problems, animal ethical mourners could situate their individual guilt into the context of structural injustices and corporate culpability: in a society built on using animal products, it is not always easy to separate oneself from all those practices.^22^

## 11 Discussion

### 11.1 Contested and contrapuntal grief

The analysis and discussion above has shown that many types of loss and grief can manifest in relation to non-human animals. The frameworks explored by Pihkala (34) in relation to ecological grief in general were all found to be relevant in relation to animal ethical mourning. In this section, we bring together major results of our discussion and present new ideas. First, we wish to introduce and discuss two new terms: “contested grief” and “contrapuntal grief.”

Contested grief refers to circumstances where the whole validity of one’s grief is questioned. There may be active efforts to de-legitimize the grief and underscore a normative stance where such grief should not exist. Whilst DG includes a lack of social support for one’s grief, contested grief is *actively* opposed.

Contested grief is most obvious in relation to farmed animals. Numerous psychological studies point out that many seek to avoid difficult moral questions related to, e.g., eating meat. The methods of such avoidance include cognitive dissonance and strategic ignorance, whereby an individual seeks to evade knowledge concerning the suffering of farmed animals (168, 169). We argue that contesting farmed animal grief is one further method of such avoidance. If an individual accepts the legitimacy of farmed animal grief, they will have to recognize the existence of animal suffering, and thereby engage with uncomfortable moral issues. Because of this, some may choose instead to actively argue that one should not grieve farmed animals – contesting farmed animal grief facilitates avoidance of animal ethical issues.

There are also cases where the object of one’s grief is another’s pleasure. We call this “contrapuntal grief,” using a term which originates in music theory: the emotions form counterpoints.

Contrapuntal grief is relevant particularly to wildlife and farmed animal grief. For example, a hunter may find enjoyment in the act of hunting, while some others grieve for the hunted animals, and their plummeting numbers. A uniquely gruesome example in regard to contrapuntal wildlife grief is a trophy hunter, whose thrill is to kill those endangered animals, whose demise others mourn.^23^ Contrapuntal farmed animal grief is especially widespread. To give an example, a meat-eater may delight in eating a burger, whilst some others mourn for the suffering caused by animal agriculture. This tension is epitomized in how, e.g., social media posts about veganism and animal rights are frequently targeted by comments proclaiming “But meat tastes good!.”^24^ In contrapuntal grief, one’s grief is not only disenfranchised or contested, but met with an emotionally antonymic response, which in our view renders wildlife and farmed animal grief exceptionally complicated.

In both contested and contrapuntal grief, one is faced by a contradiction. Whilst one is mourning for animal species or individuals, societies still continue business-as-usual in causing harm to those species and individuals [e.g., (151, 170)]. This means that the griever is surrounded by constant triggers for grief, exemplified by a vegan witnessing meat products in nearly every grocery store [e.g., (6)]. This further complicates wildlife and farmed animal grief. In particular, it makes these emotions heavy to bear: undergoing them while living in a society filled with triggers can be psychologically taxing.

### 11.2 Types of loss and grief: summarizing reflections

**Figure 7:**
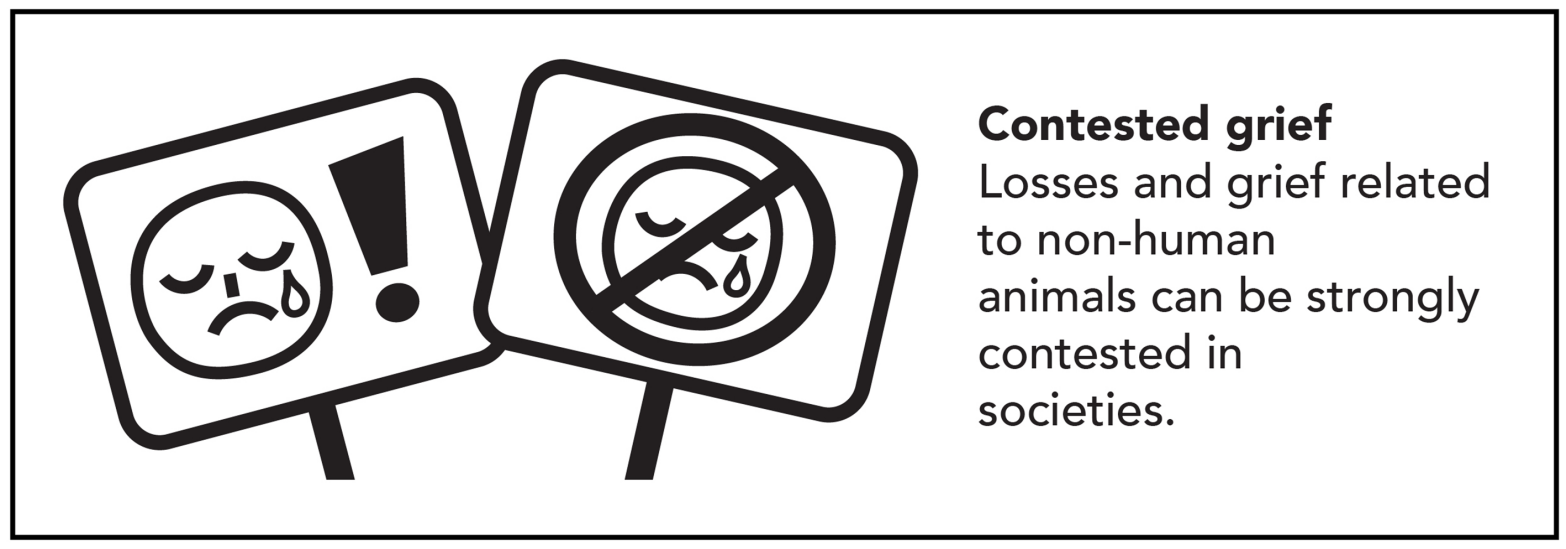
Contested grief.

**Figure 8:**
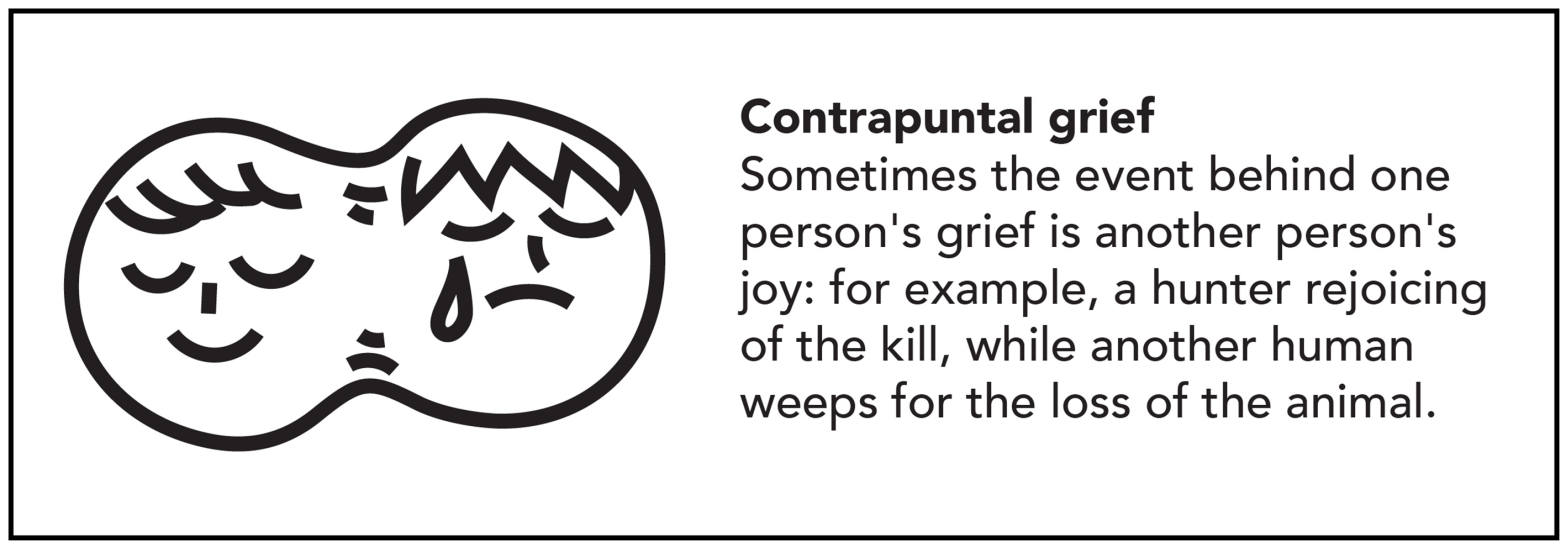
Contrapuntal grief.

**Figure 9:**
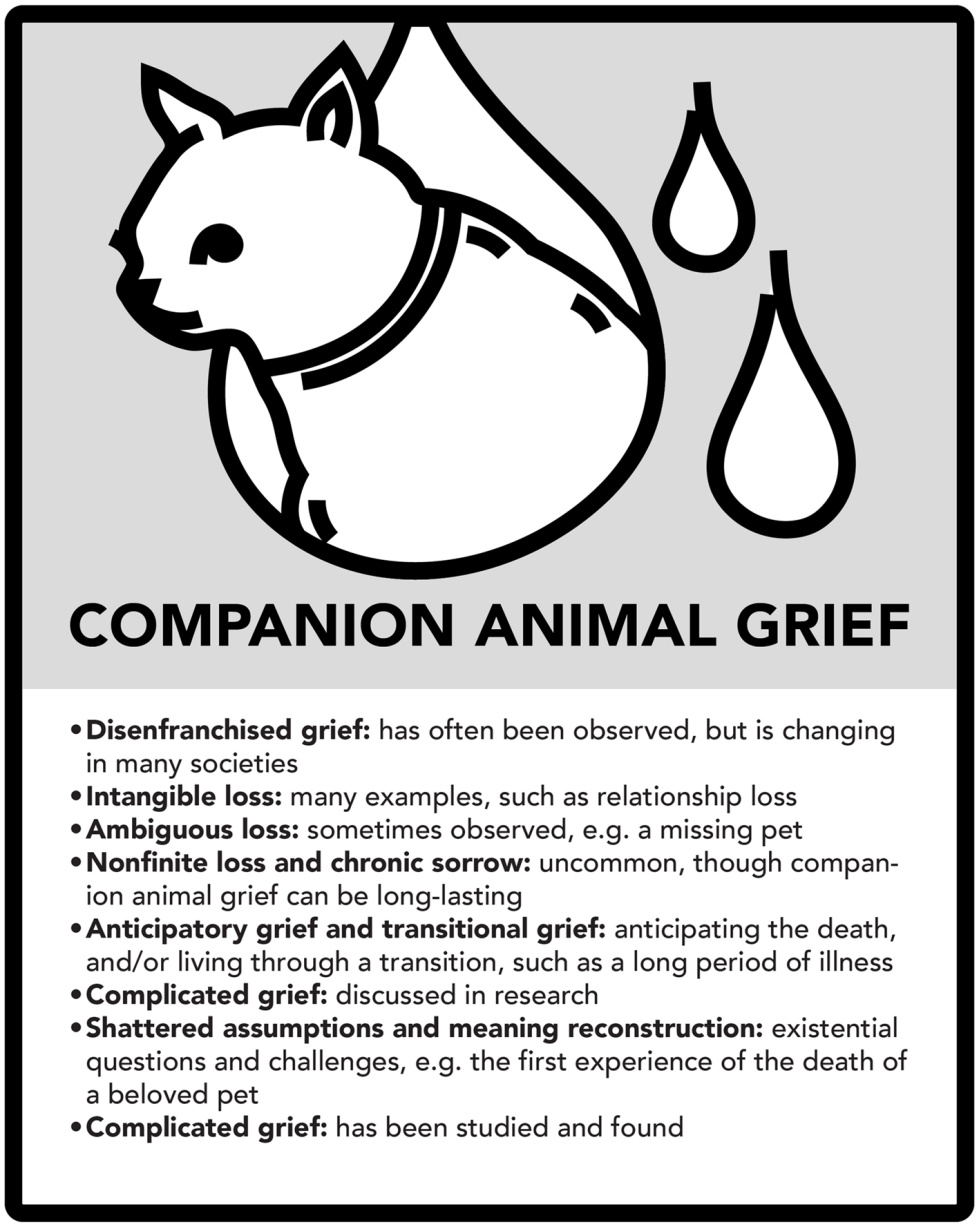
Companion animal grief.

**Figure 10:**
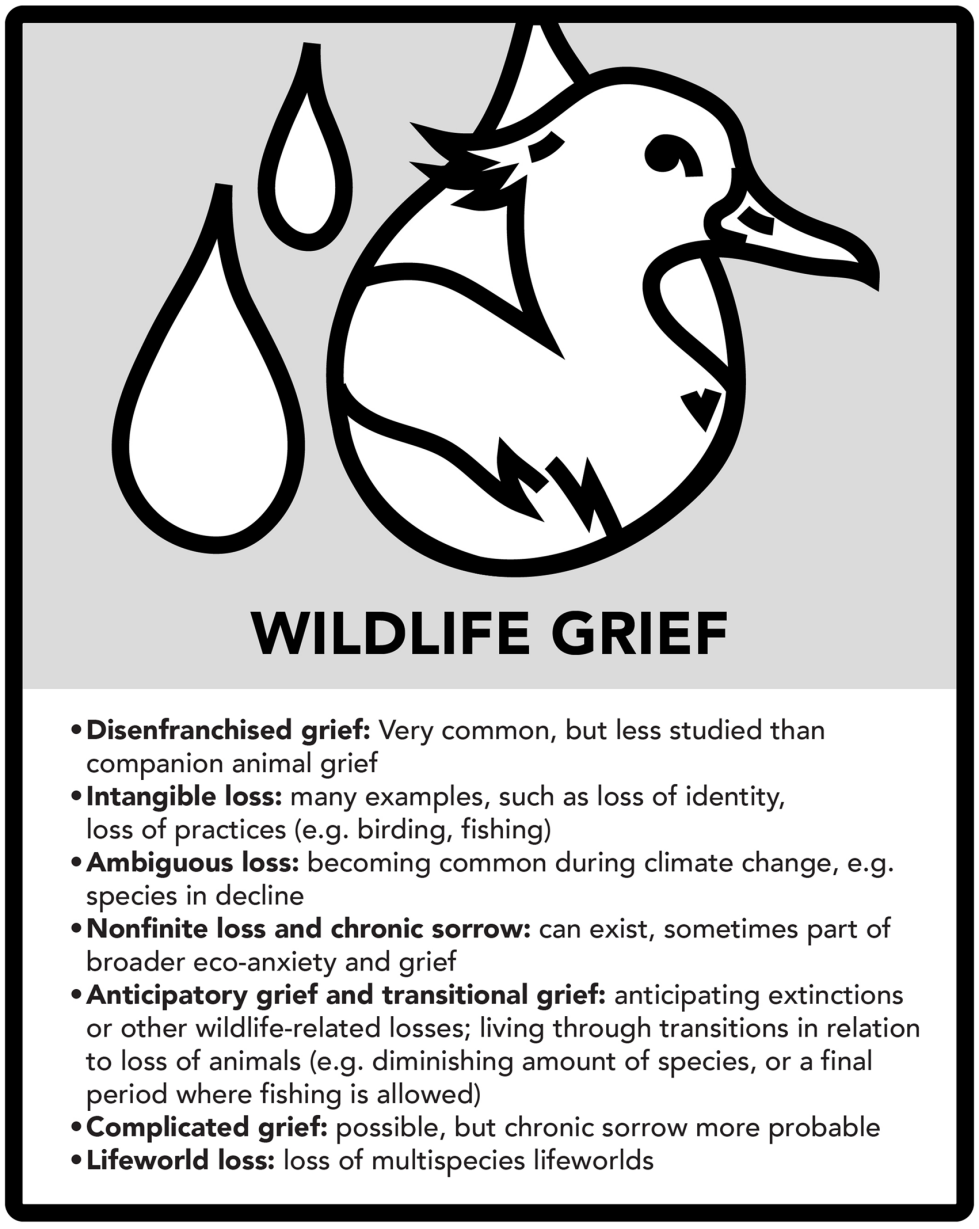
Wildlife grief.

**Figure 11:**
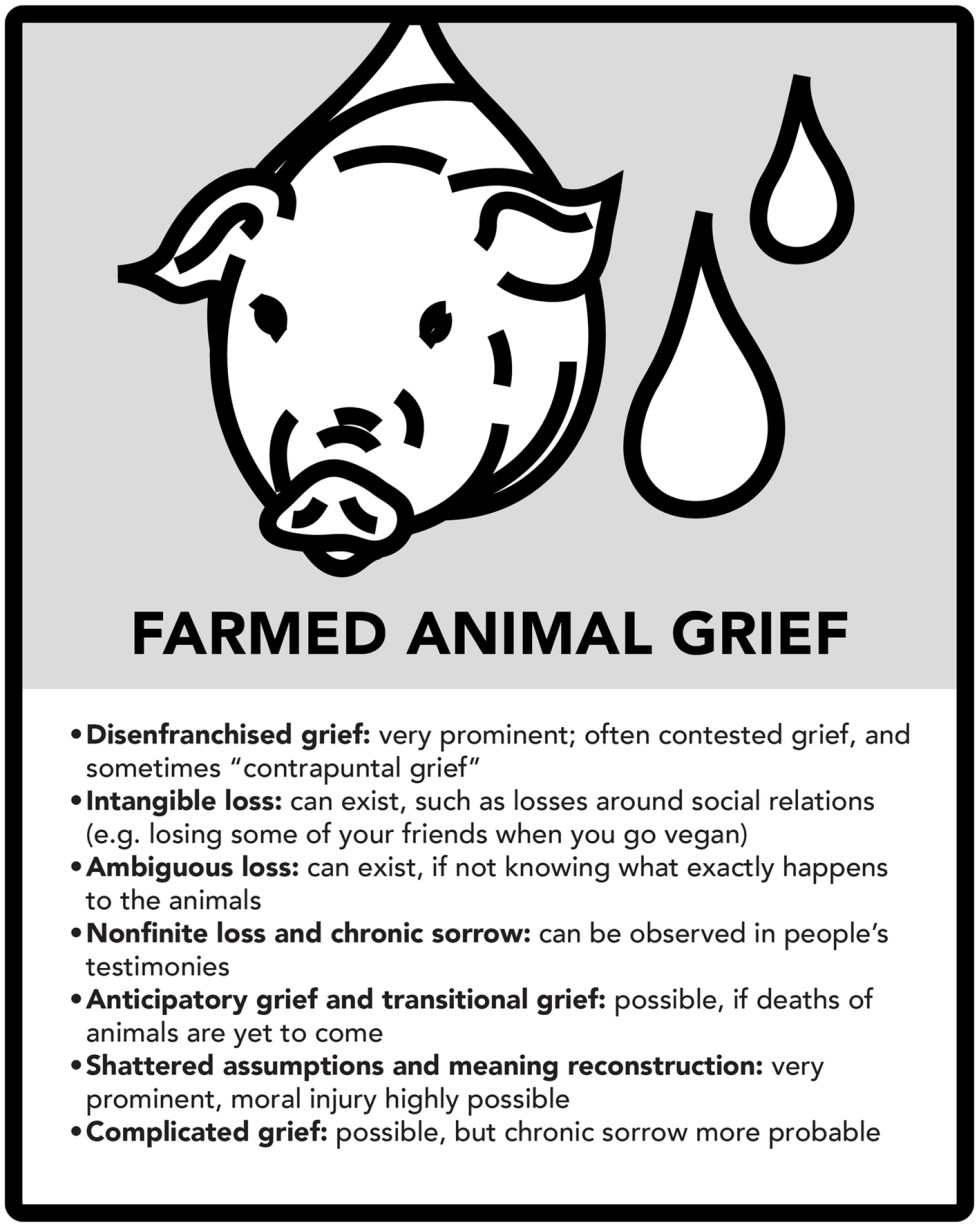
Farmed animal grief.

The analysis above shows that many kinds of loss and grief can be discerned in relation to all the three case examples. The following three figures summarize major aspects of this (see Figures 7–11).

In future research, it would be important to study animal ethical mourning empirically and make further observations about the aspects of loss and grief explored here. It should be noted that people may experience many of these griefs at the same time: for example, mourning a companion animal, mourning the disappearing bird species in the region, and mourning the fates of chicken and pigs in factory farming.

Another important topic for further research is anger and moral outrage. The role of anger is significant in grief in general [(29), pp. 20–21, 95–97], and since animal ethical mourning is so often connected with injustices and potential moral outrage, the role of anger in animal ethical mourning is evidently strong but understudied (139).^25^ In other words, grief and grievances can be strongly combined in animal ethical mourning.

Overall, various ways of coping with wildlife grief and farmed animal grief have not been much studied [for existing research, see, e.g., (82, 87, 167)], and they would deserve much more attention. It is evident that there are certain groups of people who are especially prone to difficulties in animal ethical mourning, often due to disenfranchised grief (and/or contested and contrapuntal grief), such as vegans, animal rights activists (30), animal care workers, veterinarians, veterinary nurses, and many researchers [for researchers, see, e.g., (171)]. These people would especially benefit from organizational and societal support for animal ethical mourning, and the distinctions made in this article could hopefully help to discern special needs for various types of loss and grief they face. However, the most significant societal problem is not this mourning itself, but the sufferings of non-human animals and the lack of animal ethical mourning among the wider population: the latter may sometimes be a manifestation of inhibited grief.^26^

### 11.3 Eco-anxiety and animal ethical mourning

Animal ethical mourning can be connected with a major challenge: how can people who care survive the psychological impacts of being aware? Ethical problems remain unsolved if people go into denial or disavowal in order to avoid distress. This dilemma is especially powerful in relation to children and young people.^27^ Scholars advocate for empathy, compassion, and care in the necessary process of letting youth gradually know about the state of the world, and these issues have been mostly discussed under the rubric of eco-anxiety (172–174). However, what we discuss in this article around shattered assumptions and animal ethical mourning is highly relevant for these issues. It can be especially difficult to withstand both general environmental awareness and awareness of the suffering caused to non-human animals via factory farming and animal testing in laboratories.^28^

This shows in the work of Hickman, who has interviewed children about eco-emotions and also meets youth in her therapeutic practice. She has observed that in more significant forms of eco-anxiety, high concern about animals appears regularly (175), and many children intuitively think of animals when asked to think of climate change (176). Hickman thinks that feelings of betrayal caused by adults are a deep reason for severe eco-anxiety: adults say that they care, but in political decisions they let the suffering continue (177, 178). We suspect that the shattered assumptions and moral injury caused by realization of animal rights violations can significantly strengthen these problems, and meaning reconstruction is made difficult because of disenfranchised grief and contested grief, especially in relation to farmed animal grief but also wildlife grief.

## 12 Conclusion

In this article, we have charted various types of loss and grief in what we call animal ethical mourning: grief due to moral commitment to non-human animals. Animal ethical mourning challenges human exceptionalism and it is both ethically and psychologically important, as we discuss further in another article (13). Here, we used Pihkala’s (34) categorisation of ecological loss and grief to discuss three case examples: companion animal grief (including pet grief), wildlife grief, and farmed animal grief. We brought existing studies together and offered new interpretations. Especially in footnotes, we made observations of animal-related grief by those who work with animals in captivity.

Concepts from grief theory such as intangible loss, ambiguous loss, nonfinite loss, and chronic sorrow can help to understand dynamics of animal ethical mourning. Many temporalities may be present, including anticipatory grief and transitional grief. We pointed out that there may be holistic “lifeworld losses” involved, both for humans and non-human animals. The depth of the impacts can extend to shattered assumptions about life, the world, humanity, and self. Sometimes there are elements of prolonged/complicated grief in animal ethical mourning, and various human misdeeds increase the likelihood of these. Dynamics of guilt need profound attention, as well as the broader challenge of meaning reconstruction amidst sorrows.

We discussed the prominence of disenfranchised grief in relation to animal ethical mourning, and we elaborated on various possible forms of this. In addition, we pointed out that strong social disputes and contradictions around animal rights issues result in powerful forms of disenfranchised grief. We developed two new concepts to describe these issues: Contested grief and Contrapuntal grief.

There is thus a strong need to develop more social support for animal ethical mourning. Peer groups are highly important, but there is also a need for psychological support structures and organizational development in workplaces where animals are mourned. The analysis above shows how many different aspects of loss and grief may be present in animal ethical mourning, and points towards the various resources in related grief theories for coping with them [e.g., (129, 179)]. It is possible to actively engage in meaning reconstruction and narrative re-telling in relation to animal ethical mourning (8), telling a story of empathetic people in transformation via grief. Various public ways of mourning, such as rituals and memorials, are important both ethically and psychologically (13, 44).

## Data Availability

The original contributions presented in the study are included in the article/supplementary material, further inquiries can be directed to the corresponding author.

## Footnotes

^1^Guilt features heavily also in results of research on zoo animal loss [e.g., (40)] and laboratory animal loss [e.g., (180)].

^2^Memorizing activities have happened also in certain animal research institutions (e.g., (31)] and zoos [e.g., (42)].

^3^In zoos, the animals may be originally wildlife, but now living in zoos, but many have been born there. There can be close relationships between animals and their keepers, and some dynamics of this resemble companion animal dynamics [e.g., (40)]. However, there are also many kinds of animal rights violations in zoos [see, e.g., (44)]. In laboratories and other animal research settings, strong and long emotional bonds may also be sometimes developed [e.g., (31)], but there can also be ethical violations which link the laboratory contexts with issues related to farmed animal grief [see, e.g., (43, 44)]. Both zoos and laboratories are thus contexts which can combine elements from various of our three case examples.

^4^Noted grief scholar Rando separates between Type I and Type II vicarious bereavement. In type I, a person is simply feeling empathy for another one who mourns, and sadness ensues from this process. In type II, the person’s own griefs become activated, and thus they participate in grief in a profounder way, although sometimes processing their own unfinished sorrows concerning unrelated matters (181). Pribac discusses these types in relation to farmed animal grief and observes that Type II vicarious grief may be connected to impacts on one’s assumptive world caused by realizing the amount of suffering and violence inflicted on animals (24).

^5^See https://weanimalsmedia.org/

^6^See https://aldf.org/issue/ag-gag/

^7^A telling example is one of Sainio’s respondents. She lost a long-time feline companion and contacted her employer to say that she is not sure if she is able to come to work tomorrow, because of the shock and grief. The employer terminated her employment immediately (p. 42).

^8^McDonald et al. (41) observe: “Although animal loss is a common experience for ACHPs [Animal Care and Health Personnel], only 17% of participants in our sample (16.6% of ACHPs) indicated that their zoo had a formal process or ritual that was performed following the death of an animal.” And Nageotte et al. (42) write: “Participants from a variety of organizations shared their experiences regarding their organization’s culture around grief and loss. Workplace cultures that discourage expressions of grief were mentioned frequently by participants from multiple organizations, suggesting an industry-wide norm.”

^9^Dynamics around hierarchy of species have been observed also in relation to grief about animals in laboratories and zoos, where certain species are more commonly mourned. This can lead to experiences of DG amidst those who care for species which are not publicly mourned. As one respondent tells in a study about zoos: “When large mammals or carnivores pass, the zoo [communications] will make a post about it or make some comment about it. However, I’m a bird and small-hoofstock keeper, so my animals normally do not get any recognition. I’m aware that birds die much easier than, say, a tiger, but they are still animals that we care for and bond with, and when you hear upper management, and even other keepers, say, ‘It’s just a bird’, it is very disheartening” (40, p. 10).

^10^Especially for those people working in laboratories, there can be additional complexities produced by ethical disputes about animal research in general. Proponents of practicing remembrance and mourning in animal research institutions argue that regardless of those disputes, there should be room for grief, since the animals deserve that and people function better if they can grieve their sorrows (96, 182).

^11^Here are examples: “Secondary losses often appeared as a result of animal transfer or death and added layers of complexity in how zoo professionals and volunteers adapted to the initial animal loss. The participants shared stories of walking by specific environments and missing that special animal—someone to talk with, whistle for, or share moments of deep affection. - - In addition, secondary losses associated with entire flocks or herds lost because of illness, tragedies, or dying out included a pending loss of their role, ability to work with specific animals, or their career at their facility.” (40, p. 6).

^12^For those working in animal shelters and zoos, in certain cases there may be elements of ambiguous loss generated by not knowing what happened to the animal after it was released to the wild.

^13^Similar dynamics of ambiguous loss can happen in laboratories and other animal research institutions.

^14^Moral injury has quite recently been discussed also in relation to climate change: see Weintrobe (183) and Hickman et al. (178).

^15^See, e.g., Karen Davis at https://www.upc-online.org/pp/spring2022/moral_injury_in_animal_advocates_and_nonhuman_animals.html

^16^Oliver continues: “When people first encounter vegan knowledges (that is, the violent killings and abuses of animals), anger is always at the surface, disturbing and destroying the world they thought they knew. While that anger does not dissipate, the overwhelming capacity of it has to be refused in order to continue, to instead develop and practice human-animal relationships and research ‘that celebrates our shared embodied finitude’” (139) [the quote in the last phrase is from Stanescu (6)].

^17^It is important to note that chronic sorrow is a term used differently than “chronic grief”: the latter is used by many grief researchers to describe clinically significant, complicated grief (29, pp. 142–143). On the opposite, chronic sorrow refers to long but non-pathological forms of grief.

^18^There can be long-lasting forms of companion animal grief and sometimes they fall under the category of complicated/prolonged grief, but this is not exactly the same as non-finite loss.

^19^Similar dynamics can take place among zoo workers and those working with animal research, e.g., in laboratories.

^20^In relation to pet/companion animal grief, Cleary et al. (3) report a study where “one-third of participants (23/75) found the experience left them with triggers that would renew feelings of grief for them.” They continue: “In a further study, some participants (an unspecified small number) became stuck in the experience of grief, resulting in social detachment, depression, anxiety, and poor insight into their experience,” thus manifesting complicated grief. See also Park et al. (4).

^21^These kind of dynamics have come up in research about eco-anxiety and climate anxiety/distress. See, e.g., Hickman (175, 177).

^22^Guilt, which is commonly observed in zoo and animal research laboratory workers, is there very complex because of various kinds of actual responsibility for the sufferings and deaths of the animals. Further dynamics are created by hierarchies, where some people have more power over the fates of the animals [e.g., (42)]. This whole issue merits further research and ethical discussion.

^23^See,e.g.,https://theecologist.org/2020/aug/06/trophy-hunters-threaten-endangered-animals

^24^A personal observation of the second author, who has engaged in social media advocacy for a number of years.

^25^The role of anger in relation to climate change has also recently received growing attention, and is called “climate anger.”

^26^For research on counseling in relation to pet bereavement [see (184)]. More research is needed on counseling and the two other case examples, but for reflections [see, e.g., (185–187)].

^27^For a provocative exploration of the implications of eco-anxiety for environmental education, see the article “Should we connect children to nature in the Anthropocene?” (188).

^28^For example the environmental educator and communicator, Isaias Hernandez, has discussed this intersectionality with his social media profile “QueerBrownVegan.”
